# On nodes and modes in resting state fMRI

**DOI:** 10.1016/j.neuroimage.2014.05.056

**Published:** 2014-10-01

**Authors:** Karl J. Friston, Joshua Kahan, Adeel Razi, Klaas Enno Stephan, Olaf Sporns

**Affiliations:** a The Wellcome Trust Centre for Neuroimaging, University College London, Queen Square, London WC1N 3BG, UK; b Sobell Department of Motor Neuroscience and Movement Disorders, UCL Institute of Neurology, Queen Square, London, WC1N 3BG, UK; c Department of Electronic Engineering, NED University of Engineering and Technology, Karachi, 75270, Pakistan; d Computational Cognitive Neuroscience Laboratory, Department of Psychological and Brain Sciences, Indiana University, Bloomington, IN 47405, USA

**Keywords:** Dynamic causal modelling, Effective connectivity, Functional connectivity, Resting state, fMRI, Proximity graph, Free energy, Criticality, Self-organisation, Lyapunov exponents, Bayesian

## Abstract

This paper examines intrinsic brain networks in light of recent developments in the characterisation of resting state fMRI timeseries — and simulations of neuronal fluctuations based upon the connectome. Its particular focus is on patterns or modes of distributed activity that underlie functional connectivity. We first demonstrate that the eigenmodes of functional connectivity – or covariance among regions or nodes – are the same as the eigenmodes of the underlying effective connectivity, provided we limit ourselves to symmetrical connections. This symmetry constraint is motivated by appealing to proximity graphs based upon multidimensional scaling. Crucially, the principal modes of functional connectivity correspond to the dynamically unstable modes of effective connectivity that decay slowly and show long term memory. Technically, these modes have small negative Lyapunov exponents that approach zero from below. Interestingly, the superposition of modes – whose exponents are sampled from a power law distribution – produces classical 1/**f** (scale free) spectra. We conjecture that the emergence of dynamical instability – that underlies intrinsic brain networks – is inevitable in any system that is separated from external states by a Markov blanket. This conjecture appeals to a free energy formulation of nonequilibrium steady-state dynamics. The common theme that emerges from these theoretical considerations is that endogenous fluctuations are dominated by a small number of dynamically unstable modes. We use this as the basis of a dynamic causal model (DCM) of resting state fluctuations — as measured in terms of their complex cross spectra. In this model, effective connectivity is parameterised in terms of eigenmodes and their Lyapunov exponents — that can also be interpreted as locations in a multidimensional scaling space. Model inversion provides not only estimates of edges or connectivity but also the topography and dimensionality of the underlying scaling space. Here, we focus on conceptual issues with simulated fMRI data and provide an illustrative application using an empirical multi-region timeseries.

## Highlights

• This paper presents a formal characterisation of [eigen] modes in resting state fMRI.
• It relates these to dynamical instabilities in neuronal (inferential) processing.
• It uses the equivalence between eigenmodes of functional and effective connectivity for dynamical causal modelling.

## Introduction

Recently, we described a (deterministic) dynamic causal model for resting state fMRI timeseries that tries to explain statistical dependencies or functional connectivity – as summarised with complex cross spectra – in terms of effective connectivity (Friston et al., 2014). Here, we equip the same model with an additional (graph theoretical) constraint on the effective connectivity or edges generating timeseries data. The particular symmetry constraint we consider is motivated by the recurrent nature of structural connections — and appeals to proximity graphs. The Bayesian inversion and optimisation of this model estimate both the effective connectivity and the underlying topography of the network. This topography is parameterised in terms of the location of each region in a scaling space of unknown dimension, such that the connectivity between nodes depends upon their separation.

The advantages of this model are twofold: first, it provides priors or constraints that finesse the difficult inverse problem of estimating the effective connectivity of a densely and recurrently connected graph — that generates functional connectivity. Second, it explicitly optimises the scaling space — that generates the effective connectivity. This means that one can characterise functional architectures directly in terms of their topography (relationships among nodes) in functional or scaling spaces. In principle, this approach could be used to test for differences in functional architectures between different brain states or cohorts. In this paper, we restrict ourselves to motivating the generative model, illustrating how functional topographies can be identified using Bayesian model selection and provide an illustrative proof of principle using an empirical fMRI timeseries.

### Modes, intrinsic brain networks and stability

In motivating this particular DCM, the intimate relationship between the principal modes of functional connectivity and the corresponding dynamical modes of effective connectivity becomes very apparent. We will therefore spend some time considering how functional connectivity is generated from effective connectivity — and how the emerging intrinsic brain networks or principal modes (e.g., default mode) are generated by dynamical instabilities that occur near bifurcations (Deco and Jirsa, 2012) Bifurcations are changes in the qualitative behaviour of a system as one mode of behaviour becomes unstable and yields to another. For example, a damped pendulum will eventually come to rest (at its fixed point attractor) but as the dampening decreases to zero, the attractor becomes unstable and the pendulum swings indefinitely (and its fixed point attractor becomes a periodic attractor). This loss of stability is interesting because it links intrinsic brain networks, functional connectivity, dynamical stability and self-organised criticality. We will also examine the fundaments of self-organised instability and conjecture that slowing is a necessary property of any dynamical system that shows nonequilibrium steady-state dynamics (Breakspear, 2004, Haken, 1983). These theoretical considerations are used to motivate the dynamic causal model, in which a small number of unstable (slowly decaying) modes are responsible for shaping the observed functional connectivity — and their associated intrinsic brain networks.

### From modes to graphs

In recent years, there has been an interesting convergence of graph theory and causal modelling of fMRI timeseries, particularly resting state or endogenous fluctuations (Biswal et al., 1995, Biswal et al., 1997). This convergence manifests in a number of ways; for example, the graph theoretic descriptions of adjacency matrices based upon structural or functional connectivity measures using diffusion weighted and functional MRI respectively (Bullmore and Sporns, 2009). These approaches are now widely applied within the context of the connectome (Sporns et al., 2005) and provide compelling descriptions of functional brain architectures (Power et al., 2011, Sporns, 2010). Graph theory also underlies the analysis of connectivity in several guises. For example, dynamic causal modelling of fMRI timeseries is based upon a generative model that itself is a graphical model of dependencies among different regions or nodes. Another important example is the use of multidimensional scaling and related techniques in machine learning that characterise dependencies or similarity among observations in terms of proximity graphs (Carreira-perpiñán and Zemel, 2004, Friston et al., 1996). Proximity graphs are graphs in which the connections are some well-behaved function of the distance between nodes in a scaling space that usually has to be inferred from the data. A simple example of this is (metric) multidimensional scaling also known as principal coordinate analysis, in which the (angular) proximity in scaling space is proportional to the correlation between nodes. These sorts of characterisations have a long history in the analysis of functional connectivity: one of the first applications addressed differences between the functional topography of normal and schizophrenic subjects (Friston et al., 1996). In what follows, we essentially augment a relatively simple model of fluctuations in fMRI timeseries – as summarised with their complex cross spectra – by equipping it with priors based upon the proximity graphs used in multidimensional scaling.

### Modes, graphs and modelling

The conceptual contribution of this work is to absorb constructs from dynamical systems theory and proximity graphs into the Bayesian modelling of observed timeseries. This has a number of pragmatic advantages. First, as noted above, these constructs can serve as useful constraints on the estimation of connectivity or causal structure generating statistical dependencies among observations. In many instances, estimating connectivity is a difficult inverse problem, especially when connections are reciprocal and dense. A ubiquitous example of this would be the failure of structural equation modelling to discriminate between different models with many reciprocal connections. The difficulty rests on the fact that although two structural equation models may have very different parameters (path coefficients) and may be distant in parameter space, they produce very similar data features that are close in data space. In structural equation modelling, these data features are the sample covariances among observations. By placing prior constraints on the parameters one can finesse this problem; for example, requiring the path coefficients to be the same in both directions — or by requiring them to conform to some geometric rules afforded by the location of nodes in some metric space.

We will use both of these constraints by appealing to the fact that extrinsic (long range) cortico-cortical connections are universally excitatory (mediated by glutamatergic projections) and are largely reciprocal (Markov et al., 2013). Clearly, this does not mean that the effective connectivity is always positive — because excitatory afferents could target inhibitory interneurons. However, if we make the simplifying assumption that the connection strengths are equal in both directions, we can invoke a scaling space that is equipped with a weighted (but undirected) adjacency matrix. The symmetry constraint is a necessary aspect of any proximity graph, because connectivity is a function of the distance between two nodes, which (by definition) is the same both directions.

### Proximity graphs and multidimensional scaling

We will use a multidimensional scaling space because (unlike many proximity graphs) it accommodates negative connections. Furthermore, it has a direct relationship with resting state networks or modes: resting state networks are generally defined in terms of the principal components or eigenmodes of the functional connectivity (correlation or covariance) matrix. Because these eigenmodes are unitary and orthogonal, their sum of squares is the same over the nodes of an eigenmode and the eigenmodes of a node. This means, one can plot each region on a hypersphere in an *m*-dimensional scaling space. This is known as principal coordinates analysis or metric multidimensional scaling (Friston et al., 1996). In this scaling space, the correlation between two nodes is the cosine of the angle they subtend at the centre of the sphere. This means that regions that are close together have a high functional connectivity, whereas regions on diametrically opposite sides of the sphere (e.g., the North and South Pole) are negatively correlated. Uncorrelated or functionally unconnected nodes lie halfway between (e.g., the North Pole and Equator).

The second advantage of placing graphical constraints in generative models of functional connectivity is that one can use Bayesian model comparison to ask questions about the topography of the connectivity — in terms of the dimensions of the scaling space. This is closely related to manifold learning (strictly speaking inference) procedures in machine learning that try to identify low dimensional subspaces responsible for the similarities among observed data. Examples here would include the use of principal curves (manifolds) that contain densely interconnected nodes. We will see examples of this application of subspace identification later, when comparing models based upon scaling spaces of different dimensions. This is potentially important because the dimensionality of the scaling space dictates the topography that best explains the data. Having optimised the dimension of the scaling space, the underlying functional topography is then characterised explicitly by locations within the space, as in multidimensional scaling and related clustering techniques. Furthermore, we will see later that the dimension of the scaling space can also be interpreted as the number of dynamically unstable or slow modes that dominate nonequilibrium steady-state fluctuations. It should be noted that the notions of proximity graphs and scaling spaces are used here as heuristics that make it easy to visualise dependencies in terms of relative positions. Analytically and mathematically the important attribute of scaling spaces is their dimensionality or, more simply, the number of modes or patterns needed to describe the dynamics.

### Overview

This paper comprises four sections. The first examines the formal relationship between the eigenmodes of functional connectivity — that define resting state or intrinsic brain networks, and the associated eigenmodes of effective connectivity — that define their stability. We will see that the prevalence of each mode, in terms of functional connectivity, can be related directly to its rate of decay — as defined by something called a Lyapunov exponent. This section also shows how scale free fluctuations emerge from the superposition of fluctuating modes, where a small number of nodes decay slowly. This theme is pursued in the second section that examines the basis of dynamical instability or slowing in terms of variational free energy minimisation. This formulation provides a direct representational or Bayesian interpretation of dynamical instability in terms of keeping “options open.” The third section uses the notion that nonequilibrium steady-state dynamics are generated by a small number of dynamically unstable modes to motivate a dynamic causal model of resting state fMRI data. The basic form of this model is exactly the same as previously described for explaining complex cross spectra, as sampled from multi-region fMRI timeseries. However the effective connectivity is generated under the constraints implied by a small number of unstable modes or, equivalently, from a low-dimensional scaling space. This section uses simulated data to show how Bayesian model selection can be used to identify the dimensionality of the scaling space or the number of unstable modes. We conclude by applying the Bayesian model selection to empirical data to illustrate its application in a practical setting.

## Dynamical instability and functional connectivity

In this section, we examine the relationship between effective connectivity and the functional connectivity or correlations that it generates. Our focus will be on eigenmodes and how they are conserved when considering dynamics at the level of effective connectivity and the modes of functional connectivity. The aim of this section is twofold: first, to establish the formal links between dynamical instability, slowing and modes of functional connectivity. These formal links are then used later in the dynamic causal model of resting state timeseries by furnishing empirical priors or constraints on the underlying effective connectivity matrix. The second aim is to link resting state fluctuations to scale free dynamics that characterise nonequilibrium steady-state activity.

We start with a general formulation of neuronal dynamics in terms of stochastic differential equations. These equations describe the motion or flow of hidden neuronal states that are subject to random fluctuations. The hidden states *x*(*t*) ∈ ℝ^*N*^ are then passed through an observer function to produce noisy observations *y*(*t*) ∈ ℝ^*M*^:

(1) $$\begin{array}{c} \dot{x}=f\left(x,\theta \right)+v\\ y=h\left(x,\theta \right)+w. \end{array}$$

Here, the real valued vectors *v*(*t*) ∈ ℝ^*N*^ and *w*(*t*) ∈ ℝ^*M*^ are random fluctuations in the motion of hidden states and observations respectively. A local linearization around the system's fixed point allows us to approximate neuronal dynamics with

(2) $$\begin{array}{c} \dot{x}=\nabla_{x}f\cdot x+v\\ y=\nabla_{x}h\cdot x+w. \end{array}$$

Here, we will assume that ∇_*x*_*f* is a symmetrical (negative definite) Jacobian or matrix of effective connection strengths. This means we can decompose the effective connectivity into a series of orthogonal modes or eigenvectors *μ* ∈ ℝ^*N* × *N*^, where ∇_*x*_*f* = *μ* ⋅ *λ* ⋅ *μ*^−^ and their negative eigenvalues are on the leading diagonal of *λ* ∈ ℝ^*N* × *N*^. Here, the generalised inverse *μ*^−^ = *μ*^*T*^ is simply the transpose, because we are dealing with a symmetrical Jacobian. If the Jacobian was not symmetrical, then the modes and eigenvalues would take complex values. The eigenvalues play the role of Lyapunov exponents that tell us how quickly each node decays or dissipates. One can see this by expressing the dynamics in terms of the amplitudes $\tilde{x}$ of the modes, where $x=\mu \cdot \tilde{x}$, $v=\mu \cdot \tilde{v}$ and

(3) $$\begin{array}{c} \mu \cdot \dot{\tilde{x}}=\mu \cdot \lambda \cdot \mu^{-}\cdot \mu \cdot \tilde{x}+\mu \cdot \tilde{v}\Rightarrow \\ {\dot{\tilde{x}}}_{i}=\lambda_{i}\cdot {\tilde{x}}_{i}+{\tilde{v}}_{i}. \end{array}$$

Here and throughout, we will use ~ to denote a projection onto the space spanned by the modes. Eq. (3) means that each mode will decay exponentially at a rate proportional to the real part of the eigenvalue at (in the general case) a frequency **f** proportional to the imaginary part: 2*π***f**_*i*_ = *ω*_*i*_ = Im(*λ*_*i*_). Slow dynamics correspond to (negative real) eigenvalues or Lyapunov exponents that approach zero from below. The characteristic time constants of each mode are simply the (negative) inverse of the Lyapunov exponent. This slowing is closely related to self-organised criticality and critical slowing because the system approaches a transcritical bifurcation as the exponents approach zero. A transcritical bifurcation or phase transition occurs when the real part of the eigenvalue crosses zero — leading to (local) exponential divergence of trajectories. So what would the dynamics look like from the perspective of functional connectivity? It is easy to show that when the dynamics of the system are slow in relation to the endogenous fluctuations, the covariance among the observations (assuming the number of hidden states and observations are the same) has two parts, one caused by the hidden states and the other by observation noise:

(4) Σy=∇xh⋅Σx⋅∇xhT+Σw=μ⋅γ⋅μ−γ=∇˜xh⋅Σ˜x⋅∇˜xhT+Σ˜wΣ˜x=∫0∞μ−⋅expt⋅∇xf⋅Σv⋅expt⋅∇xfT⋅μdt=∫0∞expt⋅λ⋅Σ˜v⋅expt⋅λ*dt=−Σ˜v2Reλ=Γ⋅τ.

Here, $\Gamma \cdot I=\frac{1}{2}\Sigma_{v}$ is half the covariance matrix of the random fluctuations that are assumed to be independent from node to node. As above, the ~ notation $\tilde{\Sigma }=\mu^{-}\cdot \Sigma \cdot \mu$ denotes the covariance of fluctuations of the functional modes. Here, the (negative) inverse Lyapunov exponents *τ*_*i*_ = − 1/Re(*λ*_*i*_) are time constants that reflect the instability of each mode in terms of how slowly it dissipates. The equation above says something quite intuitive: the eigenvalues $\gamma ={\tilde{\Sigma }}_{y}$ of the functional connectivity or covariance matrix are the variance or amplitude of the fluctuations of each mode. This variability has two components. The first depends upon the amplitude of neuronal fluctuations and the Lyapunov exponents or time constants, while the second is due to observation noise. This makes sense because modes with small negative exponents will decay slowly and therefore contribute much more to the observed functional connectivity.

If we could see hidden states directly such that Σ_*w*_ = 0 and ∇_*x*_*h* = *I* their covariance would be

(5) $$\begin{array}{c} \Sigma_{x}=\mu \cdot \gamma \cdot \mu^{-}=-\Gamma \cdot \mu \cdot \lambda^{-1}\cdot \mu^{-}\\ \Rightarrow \\ \nabla_{x}f=\mu \cdot \lambda \cdot \mu^{-}=-\Gamma \cdot \Sigma_{x}^{-1}\\ \gamma_{i}=-\Gamma \cdot \lambda_{i}^{-1}=\Gamma \cdot \tau_{i}. \end{array}$$

These equalities show that the variance of fluctuations in the modes is proportional to the variance of random fluctuations and the time constants (or inverse exponents). The minus sign in the first equality appears because the Jacobian is negative definite and its eigenvalues are always negative. The second equality shows there is a simple (inverse) relationship between the functional connectivity among hidden states Σ_*x*_ (if they could be observed) and the effective connectivity ∇_*x*_*f*. This is formally similar to the relationship between partial correlations and functional connectivity (Marrelec et al., 2006), where partial correlations are based on the inverse covariance matrix. The last equality reiterates the point that slow (dynamically unstable) modes dominate functional connectivity and that these are associated with eigenvalues or exponents with small negative values.

The key thing about these results is that if we know the eigenmodes and exponents of the effective connectivity matrix, then we can generate predictions of the functional connectivity and many other data features. In particular, the cross spectral density among the observations is given by:

(6) $$\begin{array}{c} g_{y}\left(\omega \right)=\nabla_{x}h\cdot g_{x}\left(\omega \right)\cdot \nabla_{x}h^{T}+g_{w}\left(\omega \right)\\ g_{x}\left(\omega \right)=K\left(\omega \right)\cdot g_{v}\left(\omega \right)\cdot K{\left(\omega \right)}^{*}\\ K\left(\omega \right)=\mathrm{FT}\left(\exp\left(t\cdot \nabla_{x}f\right)\cdot \left[t\geq 0\right]\right)=\mu \cdot \frac{1}{\mathrm{j\omega}-\lambda }\mu^{-}. \end{array}$$

Here [*t* ≥ 0] denotes Iverson brackets (that return one if the expression is true and zero otherwise) and FT(⋅) is the Fourier transform. The first equality expresses the cross spectral density as a mixture of neuronal spectra and the cross spectral density of observation noise. The transfer functions in frequency space depend upon the mapping from hidden states to observations and the eigenmodes. One can see from this expression that a large negative real eigenvalue will suppress the transfer function and cross spectral density to negligible values. In contrast, when the real part approaches zero, a Lorentzian dependency on frequency emerges, centred on *ω* = Im(*λ*). This is formally similar to the power laws associated with scale free dynamics.

### Modes and multi-Lorentzian dynamics

In fact, the superposition of cross spectral density contributions from each mode has already been proposed as a (multi-Lorentzian) model of generic 1/**f**^*α*^ spectra that characterise fluctuations in systems that are at nonequilibrium or far from equilibrium steady-state. In particular, Watanabe (2005) shows that if the characteristic time constants *τ*_*i*_ = − 1/Re(*λ*_*i*_) of the modes are sampled from a power law distribution, 1/**f**^*α*^ spectra emerge over large frequency ranges. It is easy to see how classical 1/**f** spectra arise with the following lemma:

Lemma (power law) If the correlation lengths or time constants *τ*_*i*_ = − 1/Re(*λ*_*i*_) of a dynamical system are distributed according to *p*(*τ*) ∝ *τ*^− 2^ : *τ* > *ε* (where *ε* is a small lower bound), then the spectral density of the ensuing fluctuations has a 1/**f** form.

Proof If we assume for simplicity that each mode contributes equally to the observed fluctuations *H* ⋅ *μ*_*i*_ = 1 : ∀ *i*, and the state fluctuations are independently and identically distributed *g*_*v*_(*ω*) = *I*, then their expected spectral density is given by:

(7) $$\begin{array}{c} E_{\tau }\left[g_{x}\left(\omega \right)\right]=\int_{\varepsilon }^{\infty }p\left(\tau \right)K\left(\omega \right)\cdot K{\left(\omega \right)}^{*}\mathrm{d\tau}\\ =\varepsilon \int_{\varepsilon }^{\infty }\frac{\tau^{-2}}{\omega^{2}+\tau^{-2}}\mathrm{d\tau}=\frac{\varepsilon \pi }{2\omega }={\left(\frac{\varepsilon }{4f}\right|}_{\varepsilon \to 0}\\ \int_{\varepsilon }^{\infty }p\left(\tau \right)\mathrm{d\tau}=\int_{\varepsilon }^{\infty }\varepsilon \tau^{-2}\mathrm{d\tau}=1. \end{array}$$

In other words, the spectral density shows a classical 1/**f** form □.

**Fig. 1:**
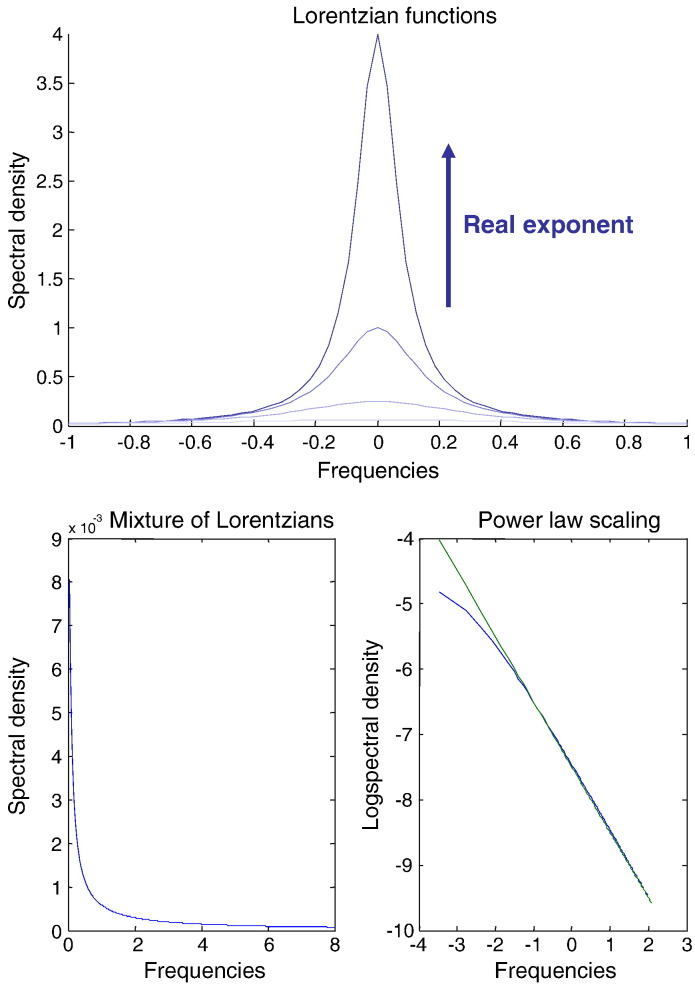
This figure illustrates the (Lorentzian) form of auto spectra induced by the eigenmodes of a dynamical system. The upper panel shows exemplar spectral densities produced by increasing the Lyapunov exponent from − 2 to − 0.25 Hz. The lower left panel shows the spectral density of mixtures of Lorentzian spectra produced by modes with Lyapunov exponents sampled from a power law distribution in the interval [− 4, − 1/128]. The plot of the logarithm of this spectral density against the logarithm of frequency should be linear — over the power law scaling regime (lower right panel). The blue line corresponds to the numerical estimate and the green line to the theoretical prediction, when the smallest real eigenvalue tends to zero. The ranges of frequencies and exponents were chosen arbitrarily for illustrative purposes.

Remarks In fact, power law scaling over ranges of frequencies emerges with the superposition of a relatively small number of modes that can be sampled from a finite interval (see also Watanabe (2005)). Fig. 1 shows an example where the time constants were restricted to the range $\left[\varepsilon =\frac{1}{256},4\right]$ and the integrals above were evaluated numerically. We are not supposing that fMRI signals necessarily show a classical power law scaling behaviour — the aim of this analysis is to show that power law scaling, indicative of nonequilibrium steady-state fluctuations, can be explained by a spectrum of Lyapunov exponents in which there are a small number of exponents that approach zero from below and a large number of large negative exponents *λ*_*i*_ ≈ − 1/*ε*, characterising modes of activity that dissipate quickly.

The lemma above assumed a particular probability distribution for the time constants that gives a 1/**f**^*α*^ form with *α* = 1. Clearly, we are not supposing that neuronal dynamics can always be described with a power law scaling with *α* = 1 or that fMRI measures responses within any power law scaling regime. However, one might safely assume that the distribution of eigenvalues is sparse with a large number of small time constants and a small number of large time constants (see next section).

We will use this assumption in the generative model below, where the unknown eigenvalues are estimated under prior beliefs that a small number will be nearly zero. These priors correspond to the hypothesis that neuronal dynamics self-organise into slow modes or patterns of activity. Crucially, if we know the eigenvalues, we also know the effective connectivity. This is because the eigenvectors of the effective connectivity ∇_*x*_*f* = *μ* ⋅ *λ* ⋅ *μ*^−^ are the eigenvectors of the (expected) sample covariance matrix Σ_*y*_ = *μ* ⋅ *γ* ⋅ *μ*^−^. One can assume this equivalence because the modes are dynamically and statistically uncoupled from each other. Having said this, there is a slight twist in that we implicitly assume that ${\tilde{\Sigma }}_{w}$ and ${\tilde{\nabla }}_{x}h$ have a diagonal form (because *γ* must have a diagonal form; see Eq. (4)). In other words, the amplitude of observation noise and haemodynamic sensitivity to neuronal responses must be roughly the same over regions before the eigenmodes of the effective connectivity can be approximated with the eigenmodes of the functional connectivity. Before considering the implications of these results for dynamic causal models of nonequilibrium steady-state fluctuations, we will address why they show the critical slowing that leads to emergence of intrinsic brain networks.

## Self-organised instability and critical slowing

In this section, we examine self-organisation using a general formulation of nonequilibrium dynamics in any system that minimises the entropy of exogenous (sensory) fluctuations that drive its internal states – either exactly by minimising self-information or approximately by minimising free energy. In fact, the following arguments apply to any system that possesses a Markov blanket that separates its internal states *x* ∈ *X* from some external states *ψ* ∈ *Ψ* (Friston, 2013). The Markov blanket contains (sensory) states *s* ∈ *S* that, for our purposes, mediate the influence of external states on internal states. This means the external states are hidden behind the Markov blanket and can be referred to as hidden states. In what follows, we first examine the basic form of the dynamics implied by exposing a self-organising system to input: in our case, exposing neuronal dynamics to sensory perturbations. These coupled systems invoke the notion of (generalised) synchronization as quantified by conditional Lyapunov exponents (CLE). This is important because the dynamics of a generalised descent on free energy have particular implications for the CLE. These implications allow us to conjecture that the local Lyapunov exponents will fluctuate around small (near zero) values, which is precisely the condition for critical slowing and the emergence of intrinsic brain networks. See Friston et al. (2012) for more details. Readers who are just interested in the dynamic causal modelling could skip this section.

### Generalised synchrony and free energy minimisation

Conditional Lyapunov exponents are normally invoked to understand synchronization between two systems that are coupled, usually in a unidirectional manner, so that there is a drive (or master) system and a response (or slave) system. The conditional exponents are those of the response system or internal states. Synchronization of chaos is the behaviour in which coupled systems exhibit identical (Barreto et al., 2003, Hunt et al., 1997) or generalised synchronization (Pyragas, 1997). The formalism of generalised synchrony means that we can consider the brain as being driven by sensory fluctuations from the environment — and that neuronal dynamics should show generalised synchrony with the sensorium. So, how does this inform self-organised criticality? The answer lies in the nature of the neuronal responses.

It is fairly simple to show that for any system that is in non-equilibrium steady-state, the flow of internal states can be expressed in terms of divergence and curl free components. However, if we limit ourselves to systems with symmetrical coupling, the divergence free component of flow disappears and we can express the dynamics of internal states as a gradient ascent on free energy as follows: for any Gibbs energy *G*(*ψ*,*s*) = − ln *p*(*ψ*,*s*) there is a free energy *F*(*s*,*x*) that describes the flow of internal states [free energy lemma: (Friston, 2013)]:

(8) $$\begin{array}{c} f_{x}\left(s,x\right)=-\Gamma \cdot \nabla_{x}F\\ F\left(s,x\right)=E_{q}\left[G\left(\psi ,s\right)\right]-H\left[q\left(\psi |x\right)\right]\\ =D\left[q\left(\psi |x\right)||p\left(\psi |s\right)\right]-\ln p\left(s\right). \end{array}$$

Here, $\Gamma =\frac{1}{2}\Sigma_{v}$ is a diffusion tensor, which – as above – is half the covariance of the random fluctuations. This (variational) free energy is a functional of a variational density *q*(*ψ*|*x*) that is parameterised by internal states. The second equality just shows that free energy can be expressed as the expected Gibbs energy minus the entropy of the variational density.

The Gibbs energy provides a probabilistic description of how sensory states are generated from hidden states. It inherits its name from statistical thermodynamics but here simply reflects the improbability of some causes and (sensory) consequences occurring together. In turn, the Gibbs energy defines the improbability, self-information or surprise − ln *p*(*s*) of any sensory state. The final equality above shows that free energy is always greater than surprise, because the (Kullback–Leibler divergence) term is non-negative. This means that when free energy is minimised with respect to the internal states, free energy approximates surprise and the conditional density approximates the posterior density over external states *q*(*ψ*|*x*) ≈ *p*(*ψ*|*s*). This is known as approximate Bayesian inference (Beal, 2003). We will call on this perspective on internal (neuronal) dynamics below, when interpreting the nature of critical slowing.

The only outstanding issue is the form of the variational density encoded by the internal states. If we admit an encoding up to second order moments, then the maximum entropy principle (Jaynes, 1957) implicit in the minimisation of free energy (Eq. (3)) requires $q\left(\psi |x\right)=N\left(x,\Sigma \right)$ to be Gaussian. This is also known as the Laplace assumption and enables one to minimise free energy with respect to the variational covariance (Friston et al., 2007).

(9) $$\partial_{\Sigma }F=0\Rightarrow \Pi =\Sigma^{-1}=\partial_{\mathrm{xx}}G\left(x,s\right)\Rightarrow F=G\left(x,s\right)+\frac{1}{2}\ln|\partial_{\mathrm{xx}}G|$$

Here, we use *G*(*x*,*s*) := *G*(*ψ* = *x*,*s*) to denote the Gibbs energy associated with the internal states. This means that one can interpret the internal states as the posterior expectations of the hidden states, while the precision *Π* = Σ^− 1^ or posterior confidence about these expectations is the curvature of the Gibbs energy.

The expression for the free energy above suggests something quite curious and remarkable. From a dynamical perspective, the free energy functional defines a landscape that directs the flow of internal (neuronal) states. This landscape is based upon the Gibbs energy but with an important difference: whenever the Gibbs energy has a high curvature the free energy is also high. This means that a free energy minimum can never have a high curvature. Heuristically, the free energy is like a mountain range in which valleys with steep sides are only found high in the mountains (c.f., hanging valleys), while lower valleys are always relatively flat (c.f., U-shaped valleys). This means, internal states – that will flow into the lower valleys – are necessarily less constrained by the free energy landscape and will show a greater sensitivity to random fluctuations. In other words, the gradient descent on free energy that characterises nonequilibrium steady-state dynamics will always flow to regions of dynamical instability, where perturbations take longer to resolve. This is the signature of critical slowing and dynamics with a long memory. This heuristic can be expressed more formally with the following lemma:

Lemma (instability) At the minima of Gibbs energy, systems at nonequilibrium steady-state are driven towards (transcritical) bifurcations as conditional Lyapunov exponents 0 ≥ *λ*_1_ ≥ *λ*_2_ ≥ … approach zero from below:

(10) $$\sum_{i}\frac{{\dot{\lambda }}_{i}}{\left|\lambda_{i}\right|}>0.$$

In other words, the proportional change in local CLE, expected under the flow, increases towards zero.

Proof Let 0 ≤ *γ*_1_ ≤ *γ*_2_ ≤ … be the real valued eigenvalues of the curvature of Gibbs energy at a minimum. The expected rate of proportional change in these eigenvalues can be expressed (by the chain rule) in terms of flow:

(11) $$\begin{array}{c} \sum_{i}\frac{{\dot{\gamma }}_{i}}{\gamma_{i}}=\partial_{t}\sum_{i}\ln\gamma_{i}=\nabla_{x}\sum_{i}\ln\gamma_{i}\cdot f_{x}\\ =-\nabla_{x}\sum_{i}\ln\gamma_{i}\cdot \Gamma \cdot \nabla_{x}F\\ F=G+\frac{1}{2}\sum_{i}\ln\gamma_{i}. \end{array}$$

The last equality follows from Eq. (9). Now, at the minimum of Gibbs energy, ∇_*x*_*G* = 0 giving

(12) $$\sum_{i}\frac{{\dot{\gamma }}_{i}}{\gamma_{i}}=-\frac{1}{2}\left(\nabla_{x}\sum_{i}\ln\gamma_{i}\right)\cdot \Gamma \cdot \left(\nabla_{x}\sum_{i}\ln\gamma_{i}\right)<0.$$

This means that – proportionally speaking – the (positive) eigenvalues shrink towards zero. So how are the eigenvalues and Lyapunov exponents related? By ignoring fourth and higher derivatives of the Gibbs energy, we can approximate the curvature of the free energy with the curvature of the Gibbs energy:

(13) $$\begin{array}{c} \nabla_{\mathrm{xx}}F=\nabla_{\mathrm{xx}}G+\nabla_{\mathrm{xx}}\frac{1}{2}\ln|\nabla_{\mathrm{xx}}G|\\ \approx \nabla_{\mathrm{xx}}G\Rightarrow \\ \nabla_{x}f_{x}=-\Gamma \cdot \nabla_{\mathrm{xx}}F\approx -\Gamma \cdot \nabla_{\mathrm{xx}}G\Rightarrow \\ \lambda \approx -\Gamma \cdot \gamma . \end{array}$$

This means that as the eigenvalues shrink to zero from above, the Lyapunov exponents approach zero from below:

(14) $$\sum_{i}\frac{{\dot{\lambda }}_{i}}{\lambda_{i}}=-\frac{1}{2}\left(\nabla_{x}\sum_{i}\ln-\lambda_{i}\right)\cdot \Gamma \cdot \left(\nabla_{x}\sum_{i}\ln-\lambda_{i}\right)<0.$$

In conclusion, a descent on free energy will be attracted to inherently unstable regions of state space with a low curvature and small local CLE □.

Remarks Because the proportional changes in CLE are dominated by CLE with small (near zero) values, the inherent drive towards zero will be more marked for the exponents of unstable modes. Note from Eq. (11) that the free energy has a logarithmic dependency on the eigenvalues and is therefore very sensitive to fluctuations in unstable modes with small eigenvalues. In short, the flow of internal states necessarily minimises the curvature of the Gibbs energy (posterior precision), thereby driving local CLE towards zero (and possibly positive) values. This produces local CLE that fluctuate at near zero values and dynamical instability or slowing. From the Bayesian inference perspective, this self-organised instability follows from the principle of maximum entropy (that generalises Laplace's principle of indifference — or Occam's razor) and reflects the intuition that, while responding sensitively to sensory perturbations, it is important to avoid overly precise or particular interpretations.

This Bayesian perspective is closely related to the motivation for metastability and critical slowing in brain dynamics that is often framed in terms of maintaining a dynamical repertoire (Breakspear, 2001, Breakspear and Stam, 2005, Jirsa et al., 1994, Kelso, 1995), particularly in relation to interpreting nonequilibrium steady-state dynamics in fMRI (Deco and Jirsa, 2012, Haimovici et al., 2013). The free energy formalism allows one to ground heuristic arguments about dynamic computations in formal arguments about representation and inference. In particular, it links the notion of flexibility, inherent in arguments about criticality and dynamic repertoires, to normative models of Bayesian inference, where critical slowing is a necessary part of free energy minimisation. This is a fundamental behaviour that reflects the need to avoid overly precise inferences to keep one's “options open.” Beyond this functional interpretation, it suggests that self-organised dynamical instability may be endemic in any (weakly mixing ergodic) system that is isolated from its external milieu by a Markov blanket.

In summary, the nonequilibrium steady-state dynamics of systems with Markov blankets can be interpreted in terms of (approximate) Bayesian inference. The ensuing flow is inherently self-destabilising because it searches out posterior expectations that have the largest margin of error (smallest posterior precision). This produces dynamical instability and slowing that is typical of systems as they approach criticality or phase transitions (that occur when the local CLE become positive producing a transcritical bifurcation). This sort of self-organised instability is closely related to, but is distinct from, chaotic itinerancy and classical self-organised criticality: chaotic itinerancy deals with itinerant dynamics of deterministic systems that are reciprocally coupled to each other (Tsuda, 2001). Here, we are dealing with systems with a skew product (master-slave) structure. However, it may be that both chaotic itinerancy and critical slowing share the same hallmark, namely, fluctuations of the local Lyapunov exponents around small (near zero) values (Tsuda and Fujii, 2004). We now return to the pragmatic problem of identifying the number and time constants of unstable modes from neuroimaging timeseries.

## Dynamic causal modelling of unstable modes

Dynamic causal modelling refers to the Bayesian inversion and selection of state-space models formulated in continuous time. This section describes a model of (resting state or activation) fMRI timeseries that is designed to identify the number of principal (unstable) modes that underlie resting state networks. This model is a standard spectral DCM (for complex cross spectra) that has been equipped with constraints on its (effective connectivity) parameters that ensure a small number of dynamically unstable modes. We will apply this model to simulated and empirical data to test the hypothesis that a small number of unstable modes best explain observed cross spectra responses.

Dynamic causal models for fMRI rest on a generative model with two components. The first is a neuronal model describing interactions in a distributed network of regions or nodes. The second maps regional activity to observed hemodynamic responses (Buxton et al., 1998, Friston et al., 2003). Here, we focus on the neuronal model, because the hemodynamic part has been described many times before, e.g., Stephan et al. (2007). The basic form of the model is a linear stochastic differential equation as in Eq. (1), where the effective connection strengths are the elements of the Jacobian. Typically, effective connectivity in fMRI falls in the range of 0.1 Hz to 1 Hz for non-trivial connections. Heuristically, these rate constants can be thought of as governing changes in the amplitude of fast (e.g., gamma band) activity (Brown et al., 2004), which waxes and wanes on the order of seconds (Breakspear and Stam, 2005). In the current DCM this effective connectivity matrix is parameterised in terms of its eigenmodes and their associated time constants.

**Fig. 2:**
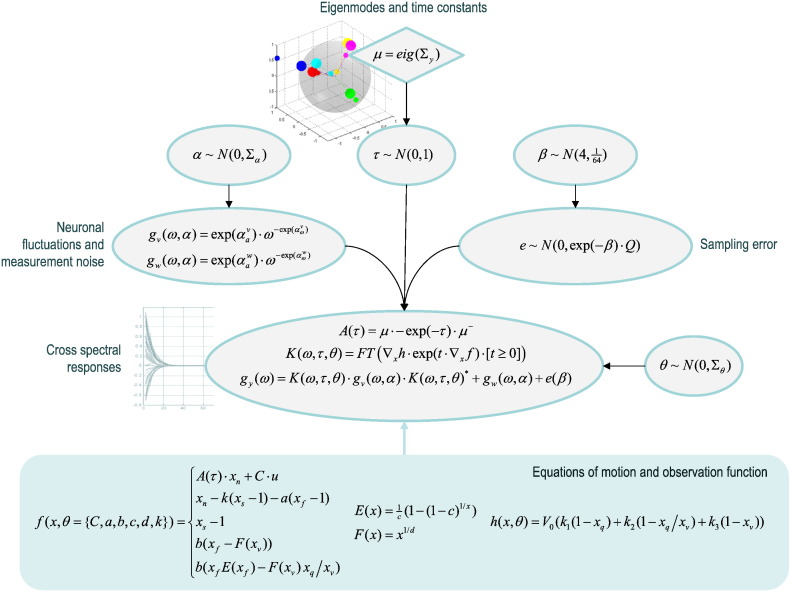
This schematic summarises the generative model for the spectral DCM described in this paper. A generative model generates observations from hidden causes. Here, we generate observed complex cross spectra by first sampling log time constants (inverse negative Lyapunov exponents) from a Gaussian distribution and using them to reconstitute an effective connectivity matrix among hidden neuronal states. When combined with regional haemodynamics (lower panel) this effective connectivity (together with other haemodynamic parameters) specifies the transfer functions mapping endogenous fluctuations to expected haemodynamic responses. The cross spectra of these responses are generated from the transfer functions given the spectral density of endogenous neuronal fluctuations and observation noise. These are generated from log amplitude and power law exponents sampled from a normal distribution. The final observations are generated with Gaussian sampling errors with a log precision sampled from a relatively informative (prior) Gaussian distribution. The key simplicity afforded by this generative model is that the eigenmodes required to generate the effective connectivity can be identified with the eigenmodes of the functional connectivity of the measured timeseries. The functions *E*(*x*) and *F*(*x*) correspond to an oxygen extraction fraction and flow functions respectively.

Fig. 2 shows the form of the generative model in terms of a Bayesian graph. A generative model is simply a model of how data are generated. In this case the data are complex cross spectra of sampled timeseries. The model starts with the spatial eigenmodes *μ* = eig(Σ_*y*_) of the sample covariance matrix. Although the number of hidden states exceeds the number of regional timeseries, we can still use the eigenmodes of the sample covariance of regional responses as proxies for the eigenmodes of hidden (neuronal) states — because there is only one neuronal state per region. The remaining hidden states model local haemodynamics, which effectively smooth or convolve the neural activity to produce a BOLD response.

The (known) eigenmodes are then combined with (unknown) Lyapunov exponents, where the associated (log) time constant *τ* ~ *N*(0,1) is drawn from a standard Gaussian prior. The resulting effective connectivity matrix *A* = *μ* ⋅ *λ* ⋅ *μ*^−^ is symmetric and negative definite because the exponents *λ* = − exp(− *τ*) are negative. The resulting effective connectivity matrix enters the equations of motion generating BOLD time series (see lower panel of Fig. 2) that specifies the transfer function in the frequency domain. This transfer function *K*(*ω*,*τ*,*θ*) depends upon haemodynamic parameters *θ* = {*C*,*a*,*b*,*c*,*d*,*k*} that specify the haemodynamic response function in each region.

This model allows one to generate predicted cross spectra by applying the transfer function to the cross spectral density of local neuronal fluctuations and adding the cross spectra of observation noise. The model is completed by specifying the likelihood of any observed data. This specification assumes that empirical cross spectra are a mixture of predicted cross spectra and some sampling error. The covariance of this sampling error is parameterised by a log-precision $\eta \sim N\left(4,\frac{1}{64}\right)$ and a fixed correlation matrix *Q* that accounts for correlations over frequencies. In principle, the form of this correlation matrix could be optimised during Bayesian model inversion; however, we find that using a fixed (autoregressive) form gives equivalent results. Note that the sampling error is distinct from the observation noise and reflects the difference between the true cross spectra and those based upon the Fourier transform of a finite timeseries. In contrast, the measurement or observation noise contributes directly to the cross spectra and – like the local neuronal fluctuations – is parameterised in terms of amplitude and power law exponents *α* for each region or node. A power law form can be motivated from studies of noise in fMRI, e.g., Bullmore et al. (2001) and underlying neuronal activity (Shin and Kim, 2006, Stam and de Bruin, 2004). In our empirical analyses later, we will use an activation study that requires the neuronal fluctuations to be supplemented with the spectral density of exogenous or experimental input *g*_*u*_(*ω*).

An interesting aspect of spectral DCM (and related analyses) is that measurement noise – that can so easily confound parameter estimates based upon the original fMRI timeseries – becomes a well-behaved component of the (predicted) spectral response. This means that high levels of noise do not render the parameter estimates less efficient; they are simply different, because these parameters include the form and amplitude of observation noise. The efficiency (posterior confidence intervals) of the estimators depends upon the sampling error that is a function of the length of the timeseries and their stationarity.

In summary, this DCM has four sets of parameters *ψ* = {*α*,*β*,*τ*,*θ*}. The first set controls the amplitude and spectral form of neuronal fluctuations and measurement noise. The second controls the precision of spectral estimation. The third parameterises the time constants of eigenmodes of neuronal activity and the fourth set parameterises regional haemodynamics. With this model, one can evaluate the likelihood of getting some spectral observations, given the model parameters *p*(*g*(*ω*)|*ψ*). The full generative model *p*(*g*(*ω*),*ψ*) = *p*(*g*(*ω*)|*ψ*)*p*(*ψ*|*m*) is then completed by specifying prior beliefs *p*(*ψ*|*m*) about the parameters, which define a particular model *m*. Because many of the parameters in these models are non-negative (scale) parameters, we generally define these priors as Gaussian distributions over ln(*ψ*). Table 1 lists the priors used in DCM for fMRI cross spectra, most of which are exactly the same as used in other DCM's for fMRI (Stephan et al., 2007).

**Table 1:** Priors on parameters (some haemodynamic priors have been omitted for simplicity).

Parameter	Description	Prior mean	Prior variance
*τ*	Log time-constants of eigenmodes	0	1
*α*	Amplitude and exponent of fluctuations noise	0	$\frac{1}{64}$
*β*	Log precision	4	$\frac{1}{64}$
*C*	Experimental input scaling	0	1
ln(*a* ⊂ *θ*)	Haemodynamic decay rate	0	*e*^− 6^
ln(*b* ⊂ *θ*)	Haemodynamic transit rate	0	*e*^− 6^

Equipped with this generative model one can now fit any observed cross spectra using standard variational Bayesian techniques (Beal, 2003). In our implementations we use variational Laplace (Friston et al., 2007) to evaluate model evidence *p*(*g*(*ω*)|*m*) and the posterior density over model parameters *p*(*ψ*|*g*(*ω*),*m*) in the usual way. In practice, we actually use both the cross spectral density and the cross covariance functions as data features.

### Simulations and face validity

**Fig. 3:**
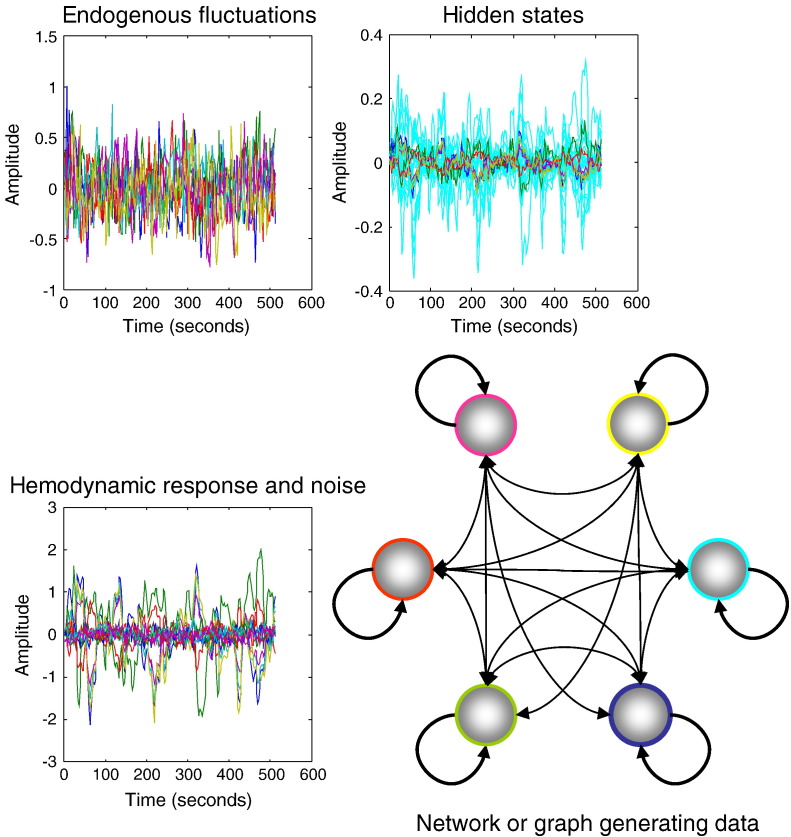
This figure shows the response of six nodes (lower right panel) over 512 (2 second) time-bins to smooth neuronal fluctuations that were generated independently in each region. These neuronal (resp. observation noise) fluctuations were generated using AR(1) processes with an autoregression coefficient of one half and scaled to a standard deviation of a quarter (resp. an eighth). The upper panels show the neuronal fluctuations (upper left panel) and consequent changes in hidden neuronal and haemodynamic (cyan) states (upper right panel) that generate the observed fMRI signal (lower left panel).

To ensure that the scheme can recover veridical estimates of effective connectivity and implicit neuronal architectures, we generated synthetic fMRI data using the equations of motion and observer function in Fig. 2. The results of these simulations are shown in Fig. 3 and show the characteristic amplitude and slow fluctuations seen in resting state time-series. This figure shows the response of six regions or nodes, over 512 (2 seconds) time-bins, to smooth neuronal fluctuations that were generated independently in each region. These temporarily correlated fluctuations (resp. observation noise) were generated using AR(1) processes with an autoregression coefficient of one half and scaled to a standard deviation of a quarter (resp. an eighth). These values were chosen to produce a maximum fMRI signal change of about 2%. The upper panels show the neuronal fluctuations and consequent changes in hidden neuronal and haemodynamic (cyan) states that generate the observed fMRI signal. Note that the fMRI signal is smoother than the underlying neuronal fluctuations, reflecting the low-pass filtering of the haemodynamic response function.

The effective connectivity generating these data was based upon the eigenmodes of the empirical data analysed below, using log time constants of *τ* = [2,1,0,− *η*,− *η*,− *η*], where we used *η* = 1 to model stable modes with a relatively fast decay or Lyapunov exponent of − exp(*η*) = − 2.72 Hz or a time constant of exp(− *η*) = 368 ms. Compare this with the principal mode that has a time constant of exp(2) = 7.4 seconds. This effectively generates data using three principal or unstable modes, which we hoped to recover using Bayesian model comparison (see below). Note that a log time constant is just the negative log decay rate, where the log decay *η*_*i*_ = ln(− *λ*_*i*_) is the log of the negative exponent of the stable modes. One can assign the same decay to fast (stable) modes because they do not contribute to the data — or at least contribute less. The definition of fast in terms of *η* is somewhat arbitrary but necessary to specify the number of remaining slow (unstable) modes.

**Fig. 4:**
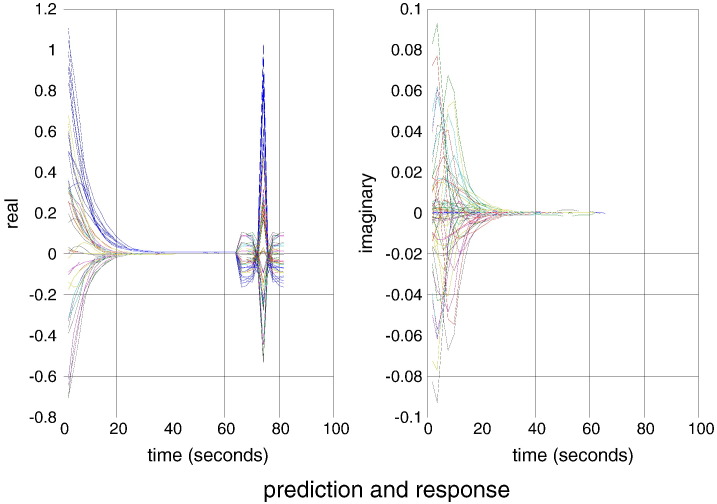
This figure shows spectral characterisation of the haemodynamic timeseries (shown in Fig. 3) in terms of sampled (dotted lines) and predicted (solid lines) responses, which are largely superimposed. The right and left panels show the imaginary and real parts of the complex cross spectra respectively, superimposed for all pairs of regions. The first half of these functions corresponds to the cross spectra, while the second half reports the associated cross covariance functions (the Fourier transform of the cross spectra).

**Fig. 5:**
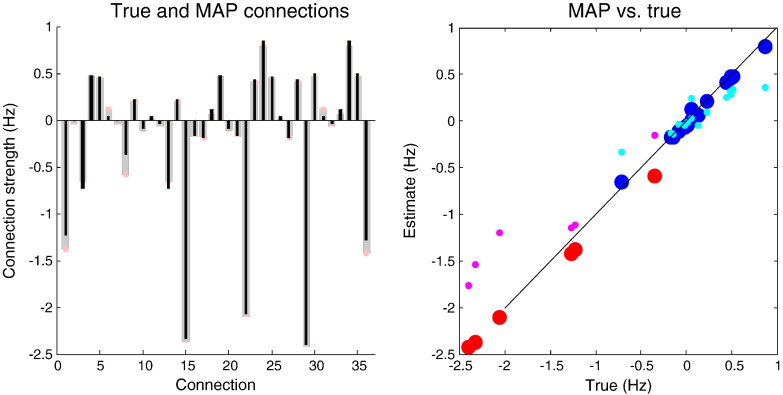
This figure reports the posterior density over the effective connectivity parameters (left panel) in terms of the posterior expectation (grey bars) and 90% confidence intervals (pink bars). For comparison the true values are superimposed (black bars). The right panel shows the same results but plotting the estimated connection strengths against their true values. The blue circles correspond to extrinsic (between-node) connections and the red circles to intrinsic (within-node) connectivity. For comparison, we have also shown the estimates from an unconstrained spectral DCM using exactly the same data and parameters (cyan and magenta circles).

The remaining model parameters were set to their usual priors and scaled by a random variate with a standard deviation of about 5%. This simulates regional variation in the haemodynamic response function. The resulting synthetic data were then used for model inversion to produce the predictions of cross spectral responses shown in Fig. 4. The sampled (dotted lines) and predicted (solid lines) cross spectra from this example can be seen in Fig. 4. The right and left panels show the imaginary and real parts of the complex cross spectra respectively, superimposed for all pairs of regions. The first half of these functions corresponds to the cross spectra, while the second half corresponds to the cross covariance functions. Note that the cross covariance functions have only real values. The agreement is self-evident with barely visible differences between the predictions and observations for the real parts. These predictions were based on the effective connectivity estimates shown in Fig. 5.

Fig. 5 shows the posterior density over the effective connectivity parameters (left panel) in terms of the posterior expectation (grey bars) and 90% confidence intervals (pink bars). For comparison, the true values used in the simulations are superimposed (black bars). The posterior estimates are remarkably accurate — largely due to the (veridical) constraints imposed on the model. Note that the posterior confidence intervals are extremely small. This should not be over-interpreted because there are profound posterior correlations between the estimators. This is due to the fact that although there are 36 effective connection strengths, we have only estimated three parameters, namely, the time constants of the three unstable modes (by fixing the prior expectations of the three stable modes to exp(− *η*) and prior variance to zero). An important point here is that the empirical eigenmodes were estimated from the sample covariance of the simulated data. The fact that we can recover such accurate estimates suggests that the empirical modes are reasonable approximations to the underlying dynamical modes.

The right panel of Fig. 5 shows the same results but this time plotting the estimated connection strengths against their true values. The blue circles correspond to extrinsic (between-node) connections and the red circles correspond to intrinsic (within node) connectivity that it is generally negative. Again, one can see the accuracy of the results with a very small root mean square error of less than 0.1 Hz. For comparison, we have also shown the estimates from an unconstrained spectral DCM using exactly the same data and parameters. These are shown as the smaller cyan and magenta circles. In this conventional model (Friston et al., 2014), stability constraints were implemented by enforcing negative intrinsic (self) connections $A_{\mathrm{ii}}=-\frac{1}{2}\exp\left(\theta_{\mathrm{ii}}\right):\forall i$ (and, in this example, symmetry constraints *A*_*ij*_ = *A*_*ji*_ = *θ*_*ij*_ : ∀ *i* ≠ *j*). One can see that the estimates with negativity constraints on the Lyapunov exponents are more accurate than those obtained with negativity constraints on the self-connections. This is because the data were generated under the former constraint. In particular, the conventional estimates of self-connections are too small, reflecting the relatively informative shrinkage priors on these parameters.

**Fig. 6:**
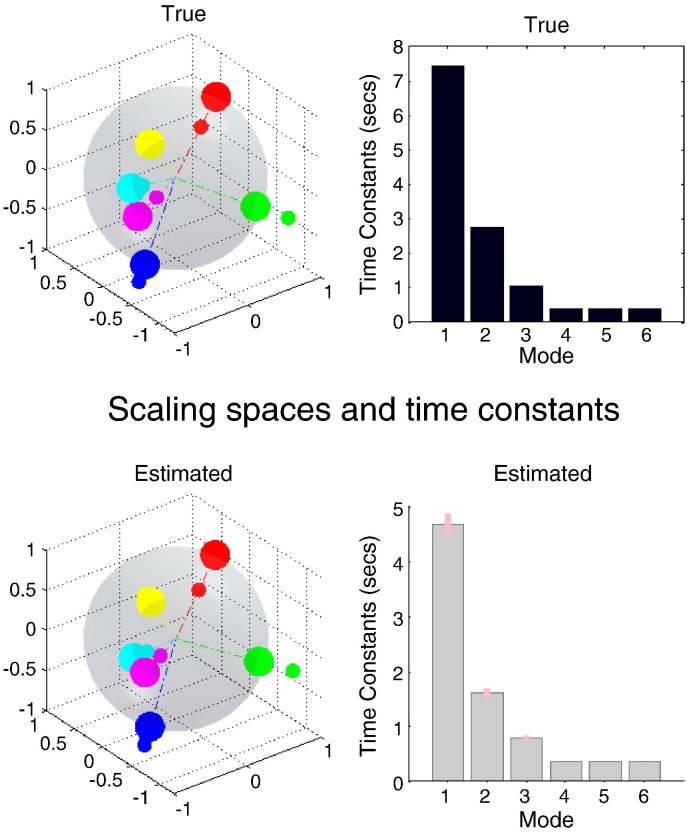
This figure shows the estimated connectivity in a (multidimensional) scaling space. The upper row corresponds to the true spatiotemporal topography used to simulate the data and the lower row shows the corresponding estimates. The topography is shown in the left panels, while the dynamics are shown on the right, in terms of the time constants (inverse negative Lyapunov exponents). The grey sphere corresponds to a unit sphere, onto which the nodes or regions (large circles) are projected, from the hypersphere on which they reside (small circles). This makes it easier to visualise their relationship to each other. The three dimensions of this scaling space correspond to the three eigenmodes of activity, with progressively decreasing time constants as shown in the right panels. The posterior expectations of the time constants are shown as grey bars and the posterior confidence intervals as pink bars.

Fig. 6 shows the connectivity in a (multidimensional) scaling space. The upper row reports the true spatiotemporal topography (used to simulate the data) and the lower row shows the corresponding posterior estimates. The topography is shown on the left, while the dynamics are shown on the right — in terms of the time constants (inverse negative Lyapunov exponents) associated with each mode or dimension of the scaling space. The grey sphere corresponds to a unit sphere, onto which the nodes (large circles) are projected, from the hypersphere on which they reside (small circles). This scaling space can be interpreted in terms of a proximity graph, where the cluster of three (magenta, cyan and yellow) regions suggests that they are strongly and positively connected. The remaining three areas are organised as anti-correlated (blue and red) regions and a disconnected (green) region. The similarity between the true and estimated topography endorses our assumption that the eigenmodes of the underlying effective connectivity (upper left) are approximately the same as the eigenmodes of the resulting functional connectivity matrix (lower left). The three dimensions of this scaling space correspond to the three eigenmodes of activity, with progressively decreasing time constants as shown in the right panels. The dynamics are clearly dominated by a slow unstable mode with a time constant of about 7 seconds. The profile of time constants estimated by the spectral DCM is very similar, although the time constants are smaller than the true values. The posterior expectations of the time constants are shown as grey bars and the posterior confidence intervals as pink bars.

The results of model inversion are explicit estimates of effective connectivity (as shown in Fig. 5) and the underlying dynamical architecture as summarised by the eigenmodes and associated time constants (as shown in Fig. 6). One might speculate that either characterisation would be an interesting candidate for characterising changes in connectivity with experimental interventions or diagnosis. The advantage of using spectral DCM in this way is that differences among conditions or groups can be characterised quantitatively in terms of dynamics. For example, the time constants have a biophysical and simple interpretation, which is a direct product of coupling that promotes critical slowing. One would imagine that the characteristic time constants of principal modes would decrease under conditions of activation — very much in the same way that electrophysiological data show a desynchronisation and loss of low frequencies in activated brain states. These are interesting considerations, particularly given the current emphasis on resting state fMRI studies (that presumably preclude highly activated brain states). In the exemplar inversion above, we assumed the true dimensionality of three principal eigenmodes. In what follows, Bayesian model comparison is used to illustrate how the number of modes can be identified.

### Bayesian model comparison

**Fig. 7:**
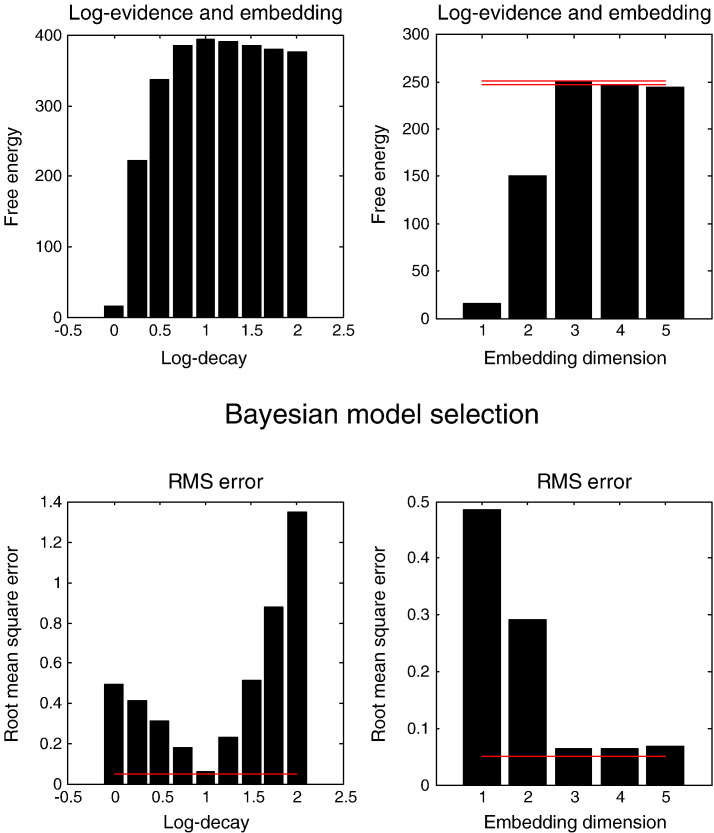
This figure shows the results of comparing models with different log exponents *η* = [0,…2] for stable modes (with three unstable modes: left panels) and the number of stable modes (with *η* = 1: right panels). The top row shows the results of Bayesian model comparison in terms of the (negative) free energy approximation to log evidence, while the lower panels report the accuracy in terms of the root mean square error, in relation to true values. The horizontal red lines in the upper right panel show the maximum log evidence (solid line) and the log evidence (broken line) that the maximum provides very strong evidence relative to. The red lines in the lower panels show an (arbitrarily) low error of 0.05 Hz.

Perhaps the greatest utility of DCM is the opportunity to compare different models or hypotheses (Penny et al., 2004). In the current context, an important aspect of the model is a number of principal or unstable (slow) modes, which is generally unknown. Identifying the order or number of modes is a common problem that is resolved using Bayesian model comparison. Fig. 7 shows the results of comparing models with different log exponents *η* = [0,…2] for stable modes (with three unstable modes: left panels) and the number of stable modes (with *η* = 1: right panels). The top rows show the results of Bayesian model comparison in terms of the (negative) free energy approximation to log evidence, while the lower panels report the accuracy in terms of the root mean square error, in relation to true values. These results show that the highest evidence is obtained when the model has the correct log exponent (log decay) for stable (dissipative) modes — at which point the root mean square error is at a minimum (around 0.05 Hz — the red line). Similarly, the model evidence peaks with the correct number of unstable (slow) modes (embedding dimension), where the accuracy is maximal. Note that the evidence for models with a greater number of unstable modes is less than the evidence of the correct model, despite the fact that these models have more degrees of freedom. In terms of Bayesian model evidence, this means that these models are slightly too complex or over-parameterised.

### An application to real data

In this final section, we apply the above analysis to an empirical data set that has been used previously to describe developments in dynamic causal modelling. These data were used to illustrate network discovery with stochastic DCM (Friston et al., 2011) and were chosen for three reasons. First, they illustrate the difference between constrained and unconstrained modelling of effective connectivity, particularly, the imposition of symmetry constraints on effective connectivity associated with the current DCM. Second, these data were elicited during an activation paradigm and allow us to show that spectral DCM can be applied to conventional studies as well as (design free) resting-state studies. Finally, although these data come from a single subject and a small number of nodes, the results can be compared directly to previous illustrative analyses.

This single subject analysis is presented to illustrate the application of this DCM and the sorts of results it furnishes. Subsequent validation papers will consider more realistic applications to resting state data — acquired in normal subjects and patients with Huntington's disease. In these analyses, we typically use between eight and 16 nodes, based upon the intrinsic brain networks of interest

### Empirical data

The data were acquired from a normal (32-year-old male) subject at 2 Tesla using a Magnetom VISION (Siemens, Erlangen) whole body MRI system, during a visual attention study. Contiguous multi-slice images were obtained with a gradient echo-planar sequence (TE = 40 ms; TR = 3.22 seconds; matrix size = 64 × 64 × 32, voxel size 3 × 3 × 3 mm). Four consecutive 100 scan sessions were acquired, comprising a sequence of ten scan blocks of five conditions. The first was a dummy condition to allow for magnetic saturation effects. In the second condition, the subject viewed a fixation point at the centre of a screen. In an attention condition, he viewed 250 dots moving away from the centre at 4.7 degrees per second and was asked to detect changes in velocity. In a no attention condition, he was asked to simply to view the moving dots. Finally, a baseline condition comprised stationary dots. The order of the conditions alternated between fixation and visual stimulation (stationary, no attention, or attention). The centre of the screen was fixated in all conditions. No overt response was required in any condition and there were no actual changes in the speed of the dots. The data were analysed using a conventional SPM analysis using three designed or experimental inputs (visual input, motion and attention) and the usual confounds. The regions chosen for network analysis were selected in a rather *ad hoc* fashion and are used simply to demonstrate procedural details.

**Fig. 8:**
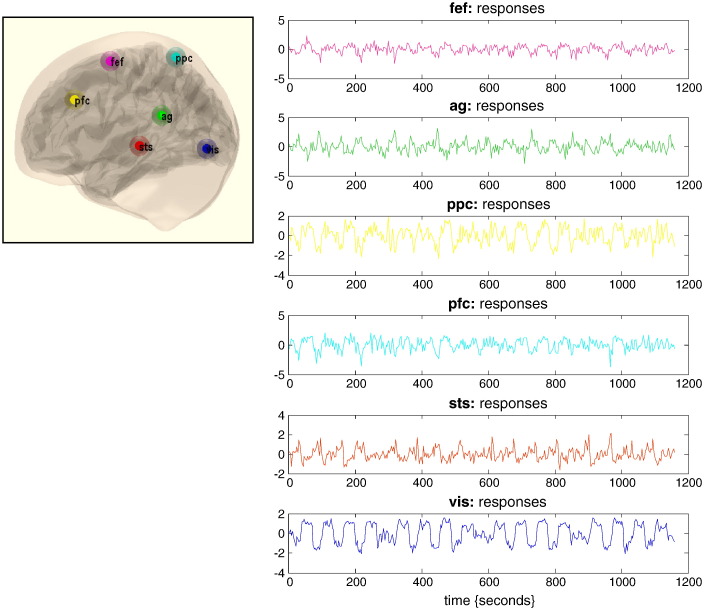
Summary of empirical timeseries used for the illustrative analysis. The timeseries (right-hand panels) from six regions show experimental effects of visual motion and attention to visual motion (see main text). These timeseries are the principal eigenvariates of nodes whose locations where identified using a conventional SPM analysis (upper left insert). See Table 2 for details.

Six representative regions were defined as clusters of contiguous voxels surviving an (omnibus) *F*-test for all effects of interest at *p* < 0.001 (uncorrected) in the conventional SPM analysis. These regions were chosen to cover a distributed network (of largely association cortex) in the right hemisphere, from visual cortex to frontal eye fields (see Table 2 for details). The activity of each region (node) was summarised with its principal eigenvariate to ensure an optimum weighting of contributions from each voxel within the ROI. In this example, one can see evoked responses in visual areas (every 60 seconds) with a progressive loss of stimulus-bound activity and a hint of attentional modulation and other fluctuations in higher regions (see Fig. 8).

**Table 2:** Regions selected for DCM analysis on the basis of an (Omnibus) SPM of the *F*-statistic testing for evoked responses. Regions are defined as contiguous voxels in the SPM surviving a threshold of *p* < 0.001 (uncorrected). The anatomical designations should not be taken too seriously because the extent of several regions covered more than one cytoarchitectonic area.

Name	Rough designation	Location (mm)	Number of (3 mm^3^) voxels
vis	Striate and extrastriate cortex	− 12 − 81 − 6	300
sts	Superior temporal sulcus	− 54 − 30 − 3	269
pfc	Prefrontal cortex	− 57 21 33	48
ppc	Posterior parietal cortex	− 21 − 57 66	168
ag	Angular gyrus	− 66 − 48 21	51
fef	Frontal eye fields	− 33 − 6 63	81

**Fig. 9:**
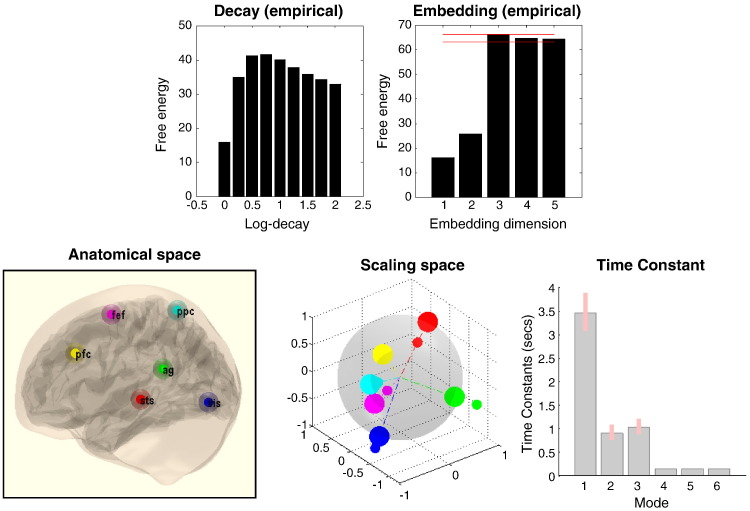
This figure reports the results of Bayesian model comparison and inversion of the empirical data. The top row uses the same format as used in Fig. 7. Here, we can see that the optimal exponent for stable modes is around 0.8 Hz, while the number of unstable modes is three. The topography of the connectivity and associated time constants are shown in the lower panels using the format of Fig. 6. In this activation study, there seems to be one dominant (slow) mode with a time constant of about 3.5 seconds. The remaining two modes have a time constant of about 1 second.

The results of Bayesian model comparison and inversion are shown in Fig. 9. The top row uses the same format as used in Fig. 7. Here, we can see that the optimal exponent for stable modes is around 0.8 Hz, while the number of unstable modes is again three. The topography of the connectivity and associated time constants are shown in the lower panels using the format of Fig. 6. The topography is identical to that in the top row of Fig. 6 — because we based the simulations on the sample covariance of the empirical data. However, we can now ascribe anatomy to the functional topography — such that the cluster of proximate nodes can be seen as belonging to association cortex, namely, prefrontal cortex, frontal eye fields and posterior parietal cortex. The anti-correlated pair of regions comprises the primary visual cortex and superior temporal sulcus. Interestingly, the angular gyrus does not seem to participate in any of these modes and is largely unconnected from all other nodes.

In this activation study, there seems to be one dominant (slow) mode with a time constant of about 3.5 seconds. The remaining two moments have a time constant of about 1 second. This suggests that the underlying fluctuations are slightly faster than one would anticipate in a resting state paradigm, perhaps reflecting the fact that these data were acquired during visual activation and switches of attentional set.

**Fig. 10:**
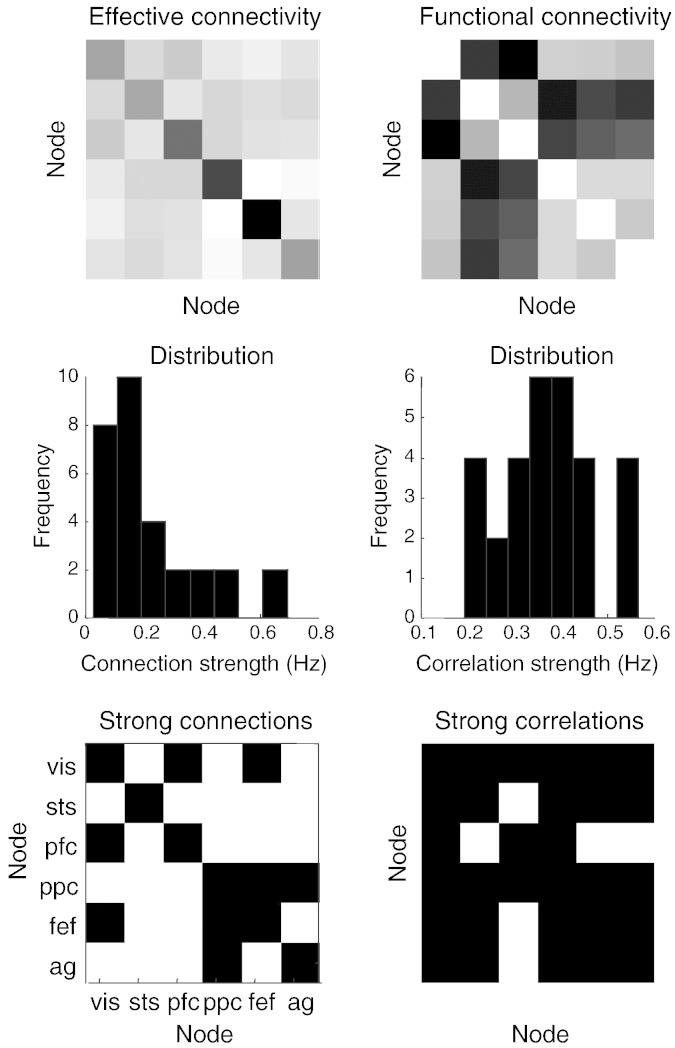
This figure shows the connectivity matrices corresponding to the proximity graph in the previous figure using an image format. The effective connectivity (upper left) is (proportional to) the inverse of the corresponding functional connectivity (upper right). The middle row shows that the distribution of effective connectivity strengths is sparser than the corresponding distribution of functional connection strengths. If we (arbitrarily) threshold the effective connectivity at 0.3 Hz and the functional connectivity at 0.3, the sparsity structure of the corresponding matrices becomes evident (lower panels). Nearly all the weak effective connections (white elements) become strong functional connections (black elements).

Fig. 10 shows the effective connectivity matrix in image format (upper left) and the corresponding functional connectivity (upper right). This functional connectivity matrix is not the conventional correlation matrix of observations — but the correlation matrix that would be seen if the hidden neuronal states could be observed directly in the absence of observation noise. The key thing to note is that the effective and functional connectivities have a very different form. In fact, as noted above, one is proportional to the inverse of the other. An important difference between effective and functional connectivity is that effective connectivity is generally much sparser. This is intuitively obvious: if there are effective connections from one node to a second — and from the second to third, these will induce functional connectivity or statistical dependencies among all three nodes. This “filling in” of a sparse effective connectivity is shown in the middle row. Here, the distribution of effective connectivity strengths is sparse, with a small number of high connections, in relation to the corresponding distribution of functional connection strengths. If we (arbitrarily) threshold the effective connectivity at 0.3 Hz and the functional connectivity at 0.3, the sparsity structure of the corresponding matrices becomes evident (lower panels). Nearly all the weak effective connections (white elements) become strong functional connections (black elements).

## Discussion

In conclusion, we have described a (spectral) dynamic causal model that could be useful in analysing resting-state studies or indeed any data reporting endogenous dynamics (e.g. sleep EEG). The motivation for this particular DCM rests upon some fundamental aspects of dynamics in coupled non-linear systems that possess a non-equilibrium steady-state. We have rehearsed some of these aspects in terms of stability analyses and the tendency of self-organised systems to critical slowing.

There are two issues that deserve special mention. The first is a practical issue highlighted by the Bayesian model comparison and assessment of (root mean square) error, in relation to true values (see Fig. 7). These results suggest that the root mean square error is very sensitive to the dissipation of stable modes. In this paper, we fixed this exponent to illustrate Bayesian model comparison; however, in routine applications this sensitivity suggests that the exponent of stable modes should be a free parameter. The second issue is more fundamental in nature. In our previous illustration of DCM using these data (Friston et al., 2011), we used a stochastic DCM to estimate the effective connectivity in the absence of constraints. A particular focus was on the asymmetries between forward and backward connections and how these define cortical hierarchies. The current (spectral) DCM precludes this sort of characterisation, because the symmetry constraints imposed upon the effective connectivity matrix require forward and backward connections to be the same. This is both a blessing and a curse: it is a blessing because it enables us to invert DCMs extremely efficiently — reducing the number of free parameters to the number of nodes. This means, in principle, one could invert extremely large DCMs in a reasonable amount of time (Seghier and Friston, 2013). Furthermore, the symmetry constraint enables a simple and graceful mapping between effective and functional connectivity (that share the same eigenmodes) and a direct interpretation in terms of undirected proximity graphs (like scaling spaces). The disadvantage is that exact symmetry constraints clearly violate known asymmetries in forward and backward extrinsic connections in the brain that – although reciprocal and excitatory – target different cortical laminae and subpopulations. Much of the available evidence suggests that backward connections target inhibitory interneurons, while forward connections target excitatory (spiny stellate) neurons in the granular layers of cortex (Bastos et al., 2012). One might argue that fMRI will be equally sensitive to pre-synaptic activity driving excitatory or inhibitory postsynaptic responses; however, the biological plausibility of undirected connectivity graphs must be, at some level, questionable. In short, the computational and conceptual advantages of the analyses considered in this paper have to be set against the implausible assumption of symmetric (undirected) coupling in the brain. As such, this form of (eigenmode) DCM could be regarded as a provisional (as if) characterisation of functional coupling that may be useful for identifying subgraphs that discriminate between different cohorts — or provide candidates for further dynamic causal modelling with (conventional) parameterisation of the effective connectivity *per se*.

Having said this, the current eigenmode DCM can, in principle, be generalised to cover asymmetric connectivity by splitting the effective connectivity into symmetric and antisymmetric components (and allowing the Lyapunov exponents to have imaginary parts). We will consider this in future work (see also the hierarchical extensions in the software note). At present, perhaps the best motivation for the current model is that it enables people to characterise resting state studies in terms of symmetrical coupling (and associated eigenmodes) and evaluate these constraints using Bayesian model comparison.

## Software note

The graphics in this paper can be reproduced using routines from the SPM academic freeware (http://www.fil.ion.ucl.ac.uk/spm/). A demonstration routine for simulating and inverting data using the current spectral DCM can be found in the DEM Toolbox (DEM_demo_modes_fMRI.m). The routine that inverts DCMs (spm_dcm_estimate.m) will automatically invoke symmetry constraints – and estimate effective connectivity in terms of eigenmodes – if the prior constraints on allowable connections (specified by a matrix) are replaced by constraints on allowable unstable modes (specified by a vector). In this paper, we have assumed that the effective connectivity is deterministically specified by its eigenmodes. This assumption can be relaxed by using the eigenmode parameterisation as a prior expectation — allowing for random variations about this expectation when estimating the effective (and now directed) connectivity. This calls for a hierarchical generative model that produces very similar results to those presented above. The specification and inversion of this hierarchical model is illustrated in DEM_demo_connectivity_fMRI.m.
